# Diastolic Dysfunction in Neonates With Hypoxic-Ischemic Encephalopathy During Therapeutic Hypothermia: A Tissue Doppler Study

**DOI:** 10.3389/fped.2022.880786

**Published:** 2022-05-25

**Authors:** Maria Jose Rodriguez, Jose Martinez-Orgado, Araceli Corredera, Irene Serrano, Luis Arruza

**Affiliations:** ^1^Division of Neonatology, Instituto del Niño y del Adolescente, Hospital Clinico San Carlos-IdISSC, Madrid, Spain; ^2^Research Methodology Unit, Hospital Clínico San Carlos-IdISSC, Madrid, Spain

**Keywords:** diastolic dysfunction, hypoxic-ischemic encephalopathy, therapeutic hypothermia, tissue Doppler, newborn, targeted neonatal echocardiography, cardiac function

## Abstract

Diastolic dysfunction often complicates myocardial ischemia with increased mortality rates. However, less is known about diastolic function after perinatal asphyxia in neonates with hypoxic-ischemic encephalopathy (HIE) during therapeutic hypothermia (TH) and rewarming.

**Aim:**

The aim of this study was to assess diastolic function with tissue Doppler imaging (TDI) in neonates with moderate–severe HIE during TH and rewarming.

**Method:**

Newborns at >36 weeks' gestation with moderate–severe HIE treated with TH were evaluated with targeted neonatal echocardiography (TNE), including TDI, within 24 h of TH initiation (T1), at 48–72 h of treatment (T2), and after rewarming (T3). These retrospective data were collected and compared with a control group of healthy babies at >36 weeks' gestation that was prospectively evaluated following the same protocol.

**Results:**

A total of 21 patients with HIE + TH and 15 controls were included in the study. Myocardial relaxation before the onset of biventricular filling was prolonged in the HIE + TH group during TH with significantly longer isovolumic relaxation time (IVRT') in the left ventricle (LV), the septum, and the right ventricle (RV). This was associated with slower RV early diastolic velocity (e') and prolonged filling on T1. Total isovolumic time (t-IVT; isovolumic contraction time [IVCT'] + IVRT') and myocardial performance index (MPI') were globally increased in asphyxiated neonates. All these differences persisted after correction for heart rate (HR) and normalized after rewarming. TDI parameters assessing late diastole (a' velocity or e'/a' and E/e' ratios) did not differ between groups.

**Conclusion:**

TDI evaluation in our study demonstrated a pattern of early diastolic dysfunction during TH that normalized after rewarming, whereas late diastole seemed to be preserved. Our data also suggest a possible involvement of impaired twist/untwist motion and dyssynchrony. More studies are needed to investigate the impact and therapeutic implication of diastolic dysfunction in these babies, as well as to clarify the role of TH in these findings.

## Introduction

Cardiovascular impairment is a frequent complication among neonates with hypoxic-ischemic encephalopathy (HIE) and has been associated with a worse neurodevelopmental outcome. Myocardial dysfunction in the context of altered cerebral blood flow autoregulation may delay brain reoxygenation after hypoxia-ischemia and consequently aggravate injury (1). Hemodynamic assessment has become a growing area of research in neonatal HIE on the grounds that a better understanding of the cardiovascular consequences of perinatal asphyxia with targeted treatment could potentially improve the outcomes. The identification of new therapeutic opportunities is of utmost importance because despite the clear benefits of therapeutic hypothermia (TH) on survival and neurological outcome, a substantial number of infants still die or remain severely affected in the long term.

In this regard, most studies have focused on the impact of perinatal hypoxia-ischemia on the systolic function of the left ventricle (LV). Only recently, the predominant vulnerability of the right ventricle (RV), and more importantly, the association of RV dysfunction with adverse neurologic outcomes, has been suggested (2, 3). Surprisingly, less attention has been paid to diastolic function in neonatal HIE, with only marginal references to it in some studies but, to the best of our knowledge, it has never been the focus of these investigations. This is striking since it is well known that myocardial ischemia in adults often results in diastolic dysfunction, even after minor myocardial damage or despite preserved LV ejection fraction (EF), and it is associated with increased mortality (4).

Diastole is a key component of heart function as adequate relaxation and ventricular filling are necessary for a normal systolic performance (5). Evaluation of diastolic function in neonates is challenging using conventional echocardiographic measurements (6). The opening and closure of the ventriculoarterial and atrioventricular valves define different mechanical events, namely, the isovolumic contraction and ejection phases during systole and the isovolumic relaxation, early, and late filling phases during diastole (7). Tissue Doppler imaging (TDI) is a relatively new technique that can provide direct measurements of myocardial velocities and timing of these myocardial events during the cardiac cycle (7) and appears to be less influenced by loading conditions and the geometry of the cardiac structures than conventional echocardiographic measurements (8). It has been demonstrated that diastolic TDI indices are strongly predictive of cardiac outcome and patient mortality, so assessment of diastolic ventricular function using TDI has been widely accepted in adult cardiology (6). Therefore, at present, TDI may be an interesting tool to assess biventricular diastolic function promptly and non-invasively (9). Although TDI is considered useful for the clinical management of some high-risk newborns, the information about its use in neonatal HIE is still scarce (10). Importantly, many studies, including TDI assessment in HIE, have been performed in babies not treated with TH (11). In fact, although TH is the standard of care for HIE newborns, the hemodynamic impact of TH and rewarming has not been fully explored yet (10, 12, 13).

The aim of this study was to assess diastolic function with TDI in newborns with moderate–severe HIE during TH and rewarming, with the hypothesis that diastolic dysfunction is another consequence of myocardial damage after HIE.

## Methods

### Study Design and Subjects

We conducted a retrospective study on newborns with moderate–severe HIE treated with TH at a tertiary neonatal intensive care unit (NICU).

**Figure 1:**
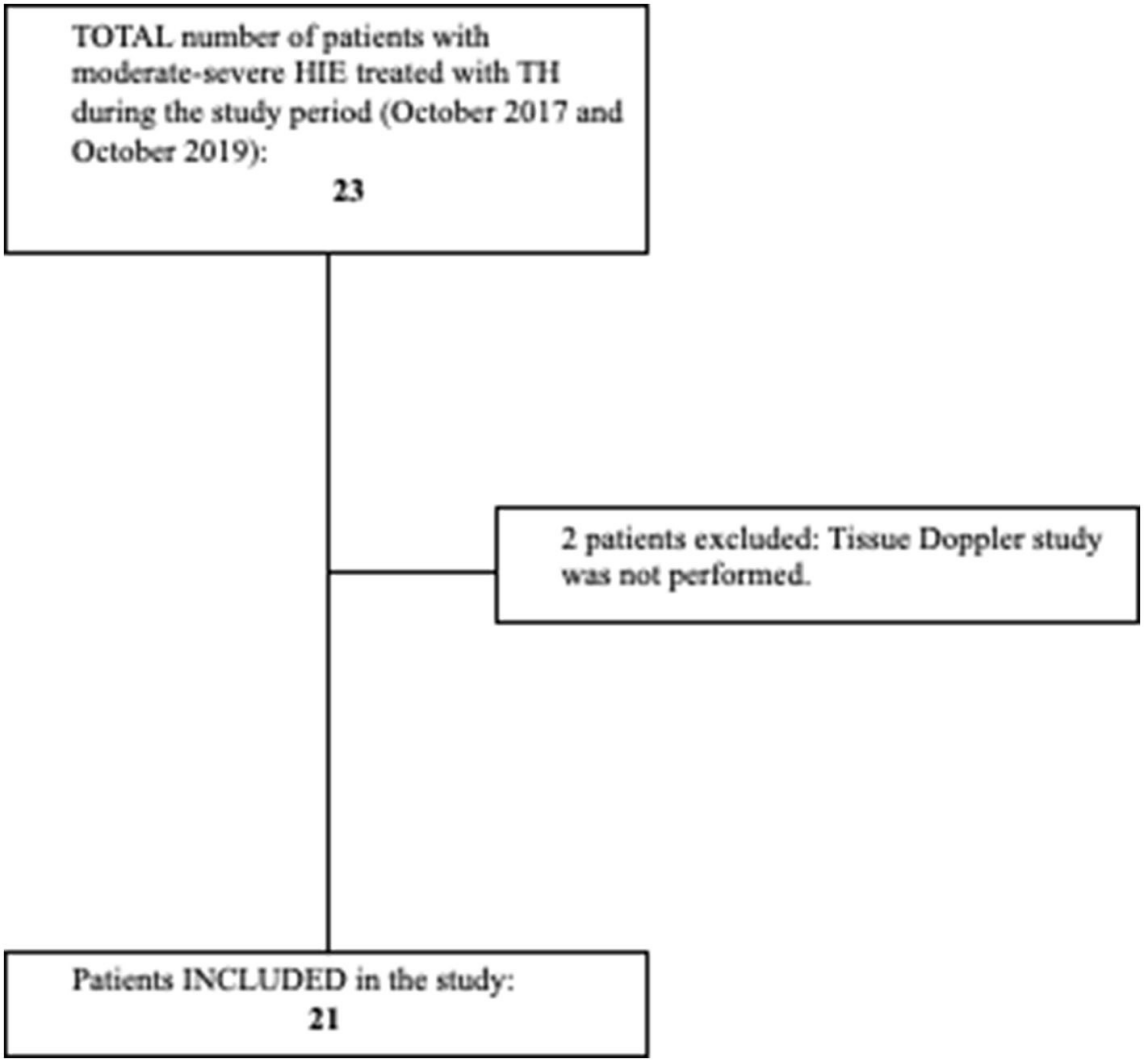
Flowchart showing patients with hypoxic-ischemic encephalopathy (HIE) included and excluded from the study. Criteria for initiation of TH: Age < 6 h, gestational age > 36 weeks, birthweight > 1,800 g. Signs of moderate–severe encephalopathy according to Sarnat score, and at least one of the following: 10-min Apgar score < 5, need for resuscitation beyond 10 min of life, cord pH or pH in the first h of life < 7.00, and base deficit ≥ 16 mmol/L in the first 60 min of life. Target rectal temperature of 33.5 ± 0.5°C was maintained for 72 h (Tecotherm Neo. Inspiration Healthcare, Leicester, UK). Rewarming was done at a rate of 0.5°C/h after cooling treatment was completed. Adapted and reprinted by permission from Springer Nature: European Journal of Pediatrics. Cerebral blood flow velocity and oxygenation correlate predominantly with right ventricular function in cooled neonates with moderate-severe hypoxic-ischemic encephalopathy (2). Copyright Springer Nature.

All patients with HIE treated with TH (HIE + TH group) admitted to the NICU at Hospital Clínico San Carlos between October 2017 and October 2019, with echocardiographic evaluations, including TDI, were eligible for enrollment. Diagnosis of congenital heart disease, decision not to provide full life support on admission, or lack of TDI assessment were considered exclusion criteria (Figure 1).

Their data were compared with a prospectively recruited control (CTL) group of healthy neonates (non-asphyxiated newborns with no signs of cardiovascular dysfunction and >36 weeks postconceptional age).

The study was approved by the local Ethics Committee. An exemption of formal consent was obtained for the inclusion of retrospective data. Parental informed consent was obtained before the enrollment of control babies.

### Clinical Data

We collected relevant demographic and clinical data of patients and controls. Gestational age, birth weight, gender, Apgar scores, cord pH, modified Sarnat score, and respiratory and cardiovascular support were recorded.

### Echocardiographic Study

The neonatal echocardiography (TNE) evaluations were performed according to our NICU protocol within 24 h of TH initiation (T1), at 48–72 h of treatment (T2), and after rewarming (T3), and at equivalent time points in the CTL group, by three different operators (i.e., MJR, AC, and LA). These studies were performed as part of the routine care of these babies to guide treatment. The use of inotropes was indicated by the attending team based on ultrasound or on clinical parameters. Images were recorded and stored in digital format for offline analysis by a single investigator (MJR). Studies were performed using a portable ultrasound device (Mindray M7; Mindray Ltd., Hamburg, Germany) with a 12-MHz transducer probe. Standard transthoracic two-dimensional, M-mode, pulsed-wave (PW) Doppler, and PW TDI images were obtained for analysis.

Conventional echocardiographic measurements were performed according to published guidelines (refer to Supplemental Material) and averaged from three to five consecutive cardiac cycles (14).

**Figure 2:**
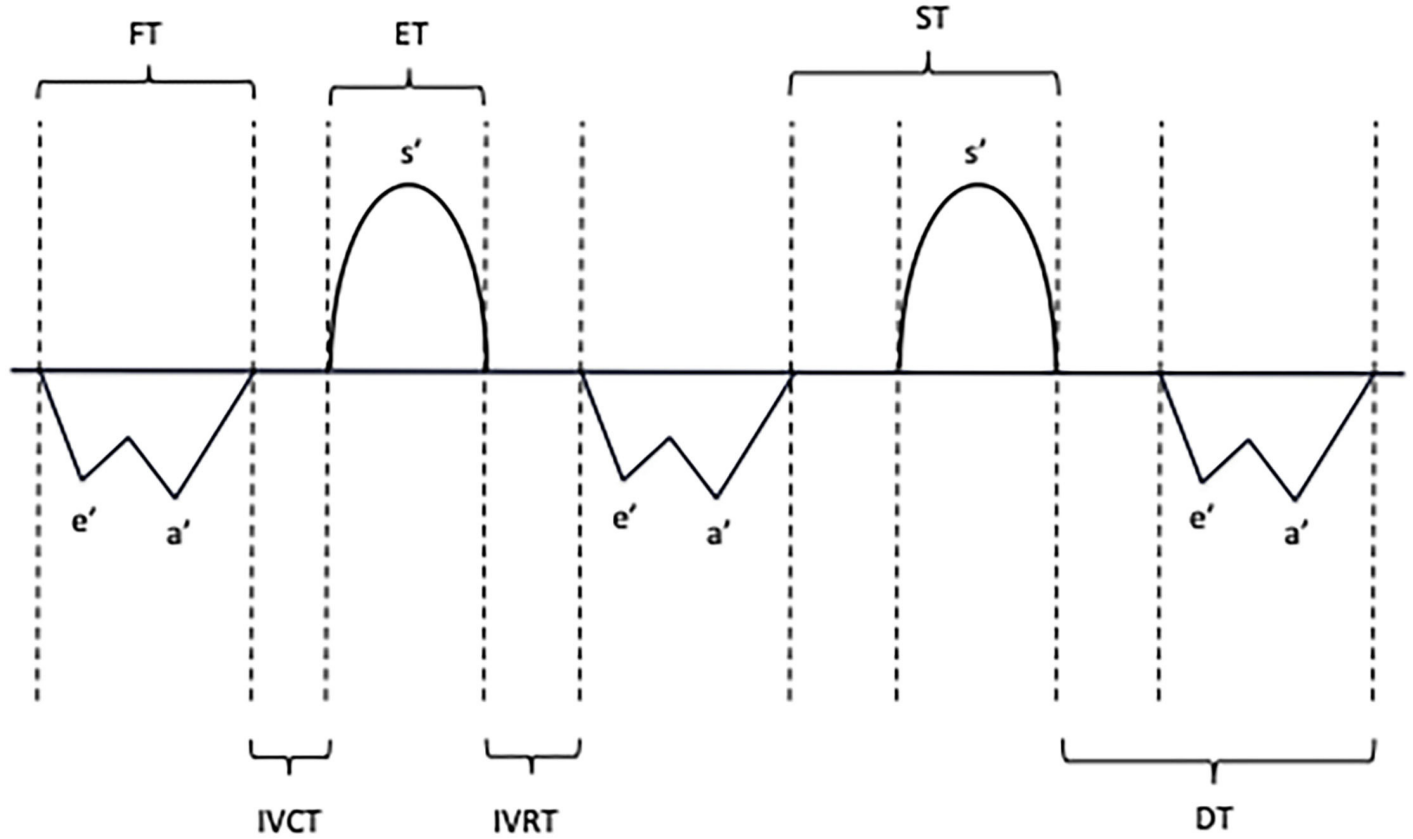
Schematic representation of systolic and diastolic myocardial velocities and time interval measurements with pulsed-wave tissue Doppler. S', Systolic velocity; E', Early diastolic velocity; A', Late diastolic velocity; IVCT, Isovolumic contraction time; IVRT, Isovolumic relaxation time; FT, Filling time; ET, Ejection time; ST, Systolic time; DT, Diastolic time.

The primary outcomes of the study were TDI myocardial diastolic velocities and time intervals. PW TDI was performed by adjusting the spectral pulsed Doppler signal filters to obtain a Nyquist limit of 15–20 cm/s and using the minimal optimal gain according to the published guidelines (7). TDI velocities were obtained from the apical four-chamber view by placing a pulsed wave Doppler sample (gate 2 mm) just below the lateral mitral annulus, the basal septal area, and the lateral tricuspid annulus (7, 15). The systolic velocity (s') and the early (e') and late (a') diastolic myocardial velocities were measured. On the PW Doppler tracings, five different time interval parameters were analyzed (Figure 2), namely, the isovolumic contraction time (IVCT', between the end of the a' wave to the beginning of the s' wave), the isovolumic relaxation time (IVRT', between the end of the s' wave to the beginning of the e' wave), the ejection time (ET', duration of the s' wave), the filling time (FT', measured from the beginning of the e' wave to the end of the a' wave), and the total isovolumic time (t-IVT'= IVCT'+ IVRT'). The e'/a', E/e' ratios, and the DTI-based myocardial performance index (MPI' = (IVCT'+IVRT')/ET') were also calculated.

Time intervals were corrected to the heart rate (HR) as calculated with the RR interval and reported as ET'/HR, IVCT'/HR, IVRT'/HR, FT'/HR, and t-IVT'/HR.

### Statistical Analysis

Considering the results of diastolic myocardial velocity measurements, for a statistic power of 90%, 1-α of 95% with d = −2, and S^2^ of 3.8, the calculated sample size was 16. The final number of babies in the control group was 15, because data from one of the babies had to be eliminated. All babies from the study group were included in the analysis. Comparison between HIE + TH and control groups was analyzed using generalized estimating equation (GEE) models with an exchangeable correlation structure. The cardiac variables were introduced in each model as dependent variables and the treatment group (TH vs. control), different time points, and the interaction term between group and time points as independent variables. The results are presented as the means and 95% confidence intervals obtained through the margins postestimation STATA command after fitting each model. The means of intragroup comparison between the treatment group (TH vs. control) in each time point and the intragroup differences, taking the time point one as reference, were made using the Bonferroni method for multiple comparisons. A value of *p* < 0.05 was accepted for statistical significance. The STATA version 15.0. software was used for the statistical analysis.

## Results

### Population Characteristics

A total of 21 patients with HIE + TH (five girls and 16 boys) were consecutively included in the study as well as 15 controls (five girls and 10 boys) (Figure 1). Tables 1, 2 summarize the characteristics of these babies. Gestational age and birth weights were similar in both groups. Cord pH and 1- and 5-min Apgar scores were significantly lower in the HIE + TH group. Upon admission, the severity of the encephalopathy was classified as stage II in 42.9% of the asphyxiated babies, according to the modified Sarnat score, whereas 57.1% were classified as stage III. The majority of patients with HIE + TH were receiving inotropic support at the time of the echocardiographic evaluations, with 66.7, 85.7, and 61.9% of the babies treated during the first, second, and third assessment, respectively, with dobutamine as the first-line treatment. None of the patients received hydrocortisone. Notably, 20 patients with HIE were discharged home and one baby died after redirection of care.

**Table 1:** General characteristics of patients included in the study.

	**Controls (*n* = 15)**	**HIE patients (*n* = 21)**	***p***
Gestational age (weeks)	38 (37–40)	39 (38–40)	0.51
Birth weight (grams)	3110 (2820–3425)	3000 (2950–3380)	0.42
Cord pH	7.28 (7.21–7.33)	6.99 (6.80–7.15)	<0.001
1^st^ min Apgar score	9 (9–9)	1.5 (0.75–2)	<0.001
5^th^ min Apgar score	10 (10–10)	3.5 (1–4)	<0.001
Modified Sarnat score	0 (100)	Stage II 9 (42.9) Stage III 12 (57.1)	
Seizures (number of patients)	0	11 (52.4)	
Age at attainment of therapeutic hypothermia (h)		4 (2.5–4.5)	
Brain MRI		Normal 12 (57.2%) Peripheral pattern 4 (19%) Central pattern 3 (14.3%) Central-peripheral pattern 2 (9.5%)	

*HIE, Hypoxic-ischemic encephalopathy. Data are expressed as median (interquartile range) or number (percentage) as appropriate. Peripheral pattern, Cortical damage; Central pattern, Basal ganglia damage*.

**Table 2:** Clinical and echocardiographic data of patients included in the study.

	***Controls*** ***(T = 1)***	***Controls*** ***(T = 2)***	***Controls*** ***(T = 3)***	***HIE*** ***(T = 1)***	***HIE*** ***(T = 2)***	***HIE*** ***(T = 3)***
HR	129 (124.5–133.5)	136 (128–146)	133 (123–137)	101 (92–124)*	119.5 (106.8–128.8)*	137 (128.5–149.5)#
Moment of echocardiography (Hours of life)	15 (11.5–17)	40 (38.5–53.5)#	81 (80–98.5)#	6 (3.8–15)	48.5 (35.8–60)#	96 (93–108.8)#
Patent ductus arteriosus (PDA)	10 (66.7%)	4 (26.7)	1 (6.7)	21 (100%)	11 (52.4%)	5 (23.8%)
Bidirectional or right to left PDA shunt	0	0	0	7 (33.3%)	5 (45.5%)	2 (40%)
Patent foramen ovale (PFO)	15 (100%)	15 (100%)	9 (60%)	21 (100%)	9 (42.9%)	9 (42.9%)
Bidirectional or right to left PFO shunt	0	0	0	5 (23.8%)	2 (22.2%)	2 (22.2%)
Inotropic support (Number and % of patients treated)	0 (0)	0 (0)	0 (0)	14 (66.7)	18 (85.7)	13 (61.9)
Dopamine Dobutamine Norepinephrine (Number and % of patients treated)	–	–	–	7 (33.3) 12 (57.1) 0	2 (9.5) 18 (85.7) 0	3 (14.3) 11 (52.4) 1 (4.8)
Dopamine Dobutamine Norepinephrine Maximum doses (mcg/kg/min) (Median IQR)	–	–	–	10 (5–16.3) 10 (5–10) 0	5 (5–5) 9 (5–10) 0	5 (5–5) 12.5 (8.8–15) 0.35 (0.35–0.35)
Inhaled nitric oxide (Number and % of patients treated)	0 (0)	0 (0)	0 (0)	1 (4.8)	5 (23.8)	5 (23.8)
Non–invasive ventilation	0 (0)	0 (0)	0 (0)	4 (19)	3 (14.3)	2 (9.5)
Invasive ventilation	0 (0)	0 (0)	0 (0)	16 (76.2)	15 (71.4)	16 (76.2)
Mean airway pressure (cmH2O) (Median IQR)	–	–	–	7 (6.7–7.5)	8 (7–10.5)	7 (6.8–8)
Sedoanalgesia: Morphine Fentanyl Other: midazolam (Number and % of patients treated)	–	–	–	21 (100) 20 (95.2) 1 (4.3) 0 (0)	21 (100) 19 (90.5) 2 (9.5) 1 (4.3)	7 (33.3) 5 (23.8) 2 (9.5) 0 (0)
Mean arterial pressure (cmH20) (Median IQR)	–	–	–	49 (46.5–57)	48 (43–54)	52 (49–62)
Troponin I (ng/mL) (Median IQR)	–	–	–	0.33 (0.13–0.52)	0.26 (0.09–0.54)	0.11 (0.05–0.32)

*HIE, hypoxic-ischemic encephalopathy;*

* *p <0.05 vs. Control (same time point);*

# *p <0.05 vs. time point 1 (within each group)*.

The results of conventional echocardiographic measurements are described in the Electronic Supplementary Material.

## Tissue Doppler Imaging

TDI results are summarized in Tables 3–5.

**Table 3:** TDI data from the left ventricle.

	***Controls (T = 1)***	***Controls (T = 2)***	***Controls (T = 3)***	***HIE+TH (T = 1)***	***HIE+TH (T = 2)***	***HIE+TH (T = 3)***
s'_LV_	5.36 (5.04–6.39)	5.9 (4.79–6.7)	6.04 (5.16–7.14)	5.24 (3.98–6.88)	5.73 (5.29–7.28)	5.82 (5.05–8.41)
e'_LV_	6.99 (5.95–7.75)	6.7 (5.78–8.11)	7.27 (6.52–8.33)	5.05 (4.34–7.62)	5.82 (4.5–6.76)	5.05 (4.66–6.81)
a'_LV_	6 (4.67–7.75)	6.52 (5.16–7.23)	7.59 (6.15–9.34)	6.25 (4.45–7.72)	7.23 (5.71–9.11)	7.47 (6.38–9)
E/ e'_LV_	7.62 (6.68–8.69)	6.99 (5.53–8.51)	6.92 (6.02–8.36)	8.35(5.22–9.52)	8.41 (6.38–9.45)	9.51 (6.89–9.98)
e'/ a'_LV_	1.15 (0.77–1.36)	0.98 (0.82–1.27)	1.01 (0.79–1.16)	0.86 (0.65–1.28)	0.76 (0.65–0.94)	0.75 (0.6–1)
IVCT'_LV_	54 (42–54)	48 (36–66)	45 (36–60)	*60 (48–84)	#54 (48–66)	#51 (39–57)
IVCT'/HR_LV_	0.43 (0.35–0.47)	0.38 (0.31–0.45)	0.35 (0.24–0.48)	*0.6 (0.48–0.68)	#0.47 (0.35–0.54)	#0.37 (0.29–0.43)
IVRT'_LV_	48 (42–54)	48 (48–60)	45 (36–60)	*84 (66–96)	*78 (72–114)	#57 (45–72)
IVRT'/HR_LV_	0.37 (0.33–0.45)	0.41 (0.33–0.5)	0.37 (0.27–0.45)	*0.84 (0.53–1.05)	*0.72 (0.56–1.08)	#0.43 (0.33–0.53)
MPI'_LV_	0.5 (0.42–0.55)	0.5 (0.45–0.59)	0.49 (0.38–0.6)	*0.72 (0.65–1.07)	*0.79 (0.61–1.26)	#0.67 (0.47–0.72)
ET${}_{{}^{\text{′}}\text{LV}}$	210 (192–216)	198 (192–210)	192 (180–204)	*180 (156–204)	*174 (150–204)	171 (159–192)
ET'/HR_LV_	1.61 (1.43–1.86)	1.56 (1.37–1.71)	1.43 (1.32–1.59)	1.47 (1.07–2.15)	1.41 (1.15–1.6)	1.25 (1.12–1.46)
FT'_LV_	204 (192–240)	228 (192–252)	195 (180–234)	222 (148–276)	*#174 (156–186)	#162 (150–186)
FT'/HR_LV_	1.57 (1.49–2.1)	1.69 (1.39–2.09)	1.56 (1.33–1.85)	1.98 (1.24–2.88)	#1.37 (1.19–1.7)	#1.27 (1.03–1.44)
t–IVT'_LV_	102 (84–108)	96 (90–114)	93 (78–114)	*144 (120–174)	*144 (114–174)	#108 (90–126)
t–IVT'/HR_LV_	0.82 (0.68–0.90)	0.78 (0.71–0.91)	0.69 (0.51–0.86)	*1.44 (0.98–1.73)	*1.09 (0.93–1.62)	#0.79 (0.75–0.93)

*HIE, hypoxic-ischemic encephalopathy; TH, therapeutic hypothermia; T1, T2, and T3, time points 1, 2, and 3, respectively; s', systolic velocity; e', early diastolic velocity; a', late diastolic velocity; IVCT', isovolumic contraction time; IVRT', isovolumic relaxation time; MPI', myocardial performance index; ET', ejection time; FT', filling time; t-IVT', total isovolumic time; HR, heart rate; LV, left ventricle.*

* *p <0.05 vs. Control (same time point);*

# *p <0.05 vs. time point 1 (within each group)*.

**Table 4:** TDI data from the interventricular septum.

	***Controls (T = 1)***	***Controls (T = 2)***	***Controls (T = 3)***	***HIE+TH (T = 1)***	***HIE+TH (T = 2)***	***HIE+TH (T = 3)***
s'_SEPTAL_	4.91 (4.3–5.51)	5.06 (4.46–5.66)	4.58 (4.43–5.41)	*3.42 (2.85–3.98)	4.3 (3.4–5.05)	#5.05 (4.13–5.38)
e'_SEPTAL_	5.41 (4.32–5.8)	5.22 (4.76–6.06)	5.09 (4.61–5.9)	4.06 (2.78–4.46)	4.43 (3.88–5.24)	4.46 (3.62–5.29)
a'_SEPTAL_	5.36 (4.67–6.39)	6.5(6.25–7.29)	6.36 (5.53–6.99)	4.7 (3.57–5.63)	*5.53 (4.41–6.25)	#6.02 (5.29–6.48)
e'/a'_SEPTAL_	0.87 (0.74–1.05)	0.82 (0.76–0.95)	0.82 (0.69–1.12)	0.84 (0.68–1.18)	0.8 (0.73–0.98)	0.7 (0.62–0.82)
IVCT'_SEPTAL_	42 (36–54)	48 (36–48)	42 (36–48)	*54 (48–96)	#48 (42–66)	#48 (42–60)
IVCT'/HR_SEPTAL_	0.35 (0.29–0.43)	0.33 (0.27–0.42)	0.32 (0.27–0.4)	*0.53 (0.32–1.03)	#0.39 (0.3–0.52)	#0.38 (0.31–0.44)
IVRT'_SEPTAL_	48 (42–54)	42 (36–54)	45 (36–54)	*78 (60–96)	*78 (54–84)	#60 (42–75)
IVRT'/HR_SEPTAL_	0.37 (0.33–0.45)	0.37 (0.29–0.49)	0.33 (0.28–0.44)	*0.65 (0.53–0.98)	*0.59 (0.46–0.79)	#0.45 (0.31–0.56)
MPI'_SEPTAL_	0.46 (0.35–0.56)	0.44 (0.38–0.51)	0.45 (0.35–0.48)	*0.72 (0.53–1.04)	#0.69 (0.55–0.8)	#0.64 (0.52–0.74)
ET'_SEPTAL_	210 (198–216)	204 (192–210)	201 (186–216)	198 (174–210)	186 (174–210)	*168 (150–186)
ET'/HR_SEPTAL_	1.65 (1.58–1.81)	1.55 (1.28–1.72)	1.51 (1.35–1.69)	1.53 (1.2–2.12)	1.48 (1.36–1.81)	1.33 (1.12–1.63)
FT'_SEPTAL_	198 (192–237)	222 (186–240)	204 (180–264)	234 (138–276)	#180 (162–198)	*#150 (132–192)
FT'/HR_SEPTAL_	1.61 (1.46–2.06)	1.62 (1.37–2.13)	1.56 (1.3–2.05)	2.09 (1.17–2.88)	#1.41 (1.28–1.64)	#1.14 (0.89–1.5)
t–IVT'/_septal_	96 (78–97)	90 (78–102)	90 (72–102)	*150 (108–186)	*#120 (102–144)	108# (96–117)
t–IVT'/HR_septal_	0.76 (0.68–0.80)	0.68 (0.61–0.82)	0.65 (0.55–0.79)	*1.32 (0.96–1.65)	*#0.99 (0.80–1.40)	0.79# (0.68–0.87)

*HIE, hypoxic-ischemic encephalopathy; TH, therapeutic hypothermia; T1, T2, and T3, time points 1, 2, and 3, respectively; s', systolic velocity; e', early diastolic velocity; a', late diastolic velocity; IVCT', isovolumic contraction time; IVRT', isovolumic relaxation time; MPI', myocardial performance index; ET', ejection time; FT', filling time; t-IVT', total isovolumic time; HR, heart rate.*

* *p <0.05 vs. Control (same time point);*

# *p <0.05 vs. time point 1 (within each group)*.

**Table 5:** TDI data from the right ventricle.

	***Controls (T = 1)***	***Controls (T = 2)***	***Controls (T = 3)***	***HIE+TH (T = 1)***	***HIE+TH (T = 2)***	***HIE+TH (T = 3)***
s'_RV_	7.13 (6.1–7.74)	6.76 (6.1–8.04)	7.21 (6.64–7.74)	5.92 (4.43–7.29)	7.29 (5.66–8.36)	#7.29 (6.7–9.08)
e'_RV_	6.99 (6.39–8.63)	7.59 (6.25–8.33)	7.87 (6.9–8.92)	*5.79 (3.87–7.11)	6.27 (5.73–7.2)	#7.33 (6.1–8.48)
a'_RV_	8.24 (7.5–9.97)	9.08 (7.57–9.67)	9.26 (7.62–10.42)	7.44 (3.84–9.67)	8.5 (7.35–10.51)	#9.97 (9.1–12.05)
E/ e'_RV_	6.16 (4.96–6.99)	6.94 (5.36–7.19)	5.8 (5.19–6.22)	6.6 (6.07–8.63)	7.07 (6.42–8.44)	6.27 (5.28–8.21)
e'/ a'_RV_	0.87 (0.74–1.05)	0.82 (0.76–0.95)	0.82 (0.69–1.12)	0.89 (0.65–1.10)	0.73 (0.63–0.90)	#0.73 (0.66–0.82)
IVCT'_RV_	36 (30–48)	36 (30–42)	33 (30–36)	*54 (42–78)	#42 (36–60)	*#48 (42–54)
IVCT'/HR_RV_	0.29 (0.24–0.33)	0.26 (0.23–0.32)	0.26 (0.22–0.3)	*0.48 (0.31–0.75)	#0.36 (0.27–0.51)	#0.36 (0.26–0.39)
IVRT'_RV_	48 (42–48)	48(30–54)	36 (30–48)	*78 (72–108)	*72 (54–108)	#48 (36–60)
IVRT'/HR_RV_	0.37 (0.33–0.43)	0.34 (0.26–0.44)	0.29 (0.22–0.35)	*0.78 (0.58–1.01)	*#0.61 (0.42–0.91)	#0.35 (0.26–0.48)
MPI'_RV_	0.38 (0.36–0.45)	0.38 (0.32–0.42)	0.33 (0.3–0.38)	*0.7 (0.63–0.88)	*0.69 (0.42–1.04)	#0.52 (0.36–0.64)
ET'_RV_	216 (198–234)	222 (210–234)	213 (204–222)	186 (174–222)	*192 (174–204)	*180 (162–198)
ET'/HR_RV_	1.72 (1.55–2.02)	1.66 (1.58–1.88)	1.64 (1.46–1.78)	1.72 (1.37–2.4)	1.59 (1.38–1.8)	#1.3 (1.12–1.51)
FT'_RV_	195 (174–237)	210 (180–282)	204 (192–246)	246 (174–282)	*#174 (156–198)	*#150 (126–174)
FT'/HR_RV_	1.55 (1.35–2.02)	1.57 (1.3–2.13)	1.54 (1.3–2.03)	*2.59 (1.4–3.03)	#1.28 (1.19–1.56)	*#1.16 (0.77–1.36)
t–IVT'_rv_	84 (78–90)	84 (72–90)	72 (60–84)	*150 (114–168)	*#120 (90–168)	93# (78–114)
t–IVT'/HR_rv_	0.69 (0.60–0.73)	0.61 (0.52–0.76)	0.60 (0.44–0.62)	*1.40 (0.91–1.72)	*#1.04 (0.74–1.40)	0.68# (0.53–0.87)

*HIE, hypoxic-ischemic encephalopathy; TH, therapeutic hypothermia; T1, T2, and T3, time points 1, 2, and 3, respectively; s', systolic velocity; e', early diastolic velocity; a', late diastolic velocity; IVCT', isovolumic contraction time; IVRT', isovolumic relaxation time; MPI', myocardial performance index; ET', ejection time; FT', filling time; t-IVT', total isovolumic time; HR, heart rate; RV, right ventricle.*

* *p <0.05 vs. Control (same time point);*

# *p <0.05 vs. time point 1 (within each group)*.

## Systolic Myocardial Velocities

Left ventricular (LV) myocardial systolic velocities were similar in both groups at all time points. In contrast, septal s' was decreased in patients with HIE + HT in T1 compared to controls and increased significantly after rewarming. RV s' did not differ from controls at any time point but it increased significantly in asphyxiated babies after rewarming.

## Diastolic Myocardial Velocities

The LV diastolic myocardial velocities were similar in both groups, without differences for early (e'_LV_) and late (a'_LV_) diastolic velocities, as well as E/e'_LV_ and e'/a'_LV_ ratios. Septal a' was significantly slower at T2 in asphyxiated neonates and increased significantly after rewarming, but e'_SEPTAL_ and e'/a'_SEPTAL_ did not differ between groups or time points. Regarding RV diastolic velocities, e' was significantly slower at the first assessment in the HIE + TH group and improved after rewarming. However, no differences were found for a'_RV_, E/e'_RV_, or e'/a'_RV_ between groups and time points except for a significant increase in a'_RV_ and e'/ a'_RV_ after rewarming compared to baseline values in patients with HIE.

## Time Intervals

*Isovolumic Contraction Time*. LV, RV, and septal IVCT' were prolonged in the HIE + TH group in T1 compared to controls. This difference was significant at this time point even after correction for HR. IVCT' shortened significantly in T2 and T3 in asphyxiated neonates so that differences with the control group disappeared, except for the RV where it persisted significantly prolonged after rewarming. However, when corrected for HR, this difference in the RV was no longer significant.

*Isovolumic Relaxation Time*. Similarly, IVRT' was longer in the HIE + TH group at T1 and T2 in the LV, RV, and septum, and decreased significantly after rewarming. These differences remained significant after correction for HR.

*Myocardial Performance Index*. LV and RV MPI' were increased in asphyxiated babies in T1 and T2, compared to controls, and normalized after rewarming. Similarly, septal MPI' was increased at T1 in these groups but improved in subsequent assessments.

*Ejection and Filling Times*. Regarding the duration of ET', ET'_LV_ was significantly shorter in T1 and T2, ET'_SEPTAL_ was shorter after rewarming, and ET'_RV_ was shorter in T2 and T3, in asphyxiated neonates as compared to controls, but none of these differences persisted after correction for HR. LV, septal, and RV FT' were significantly longer in infants with HIE at T1 compared to values at T2 and after rewarming, a difference that remained statistically significant after correction for HR. Moreover, when corrected for HR, FT'_RV_ at the first assessment was also longer in the HIE + TH group compared to controls. In contrast, although TDI-measured FT' in the LV at T2, in the septum at T3, and in the RV at T2 were significantly shorter in the HIE + TH group compared to controls, these differences disappeared after controlling for HR. However, FT'_RV_ was shorter in asphyxiated babies compared to controls after rewarming, with and without correction for HR.

*Total Isovolumic Time*. t-IVT (t'-IVT = IVCT' + IVRT') was significantly longer in LV, septum, and RV in babies with HIE during TH compared to controls, with a significant decrease in T2 and after rewarming.

## Discussion

In this retrospective study, we demonstrated the existence of a significant diastolic dysfunction in cooled neonates with moderate–severe HIE that improves progressively during treatment and normalizes after rewarming. TDI evaluation in our study showed a pattern of early diastolic dysfunction during TH, whereas late diastole seemed to be preserved.

This is one of the first studies specifically assessing biventricular diastolic function using TDI during TH and rewarming. We observed an increase in MPI' in both ventricles and the septum during TH that normalized after rewarming. Increased MPI is one of the most consistent echocardiographic findings in studies on HIE and reflects an alteration of combined systolic and diastolic function (16–19). In this context of global myocardial dysfunction, most investigations have focused on LV systolic function (11, 12, 20, 21), and less attention has been paid to RV function or diastolic performance. However, in high-risk newborns, diastolic heart failure may precede systolic failure and latent RV dysfunction may be present before LV failure appears (22). Newborns may be particularly vulnerable to diastolic dysfunction after HIE because, due to the relatively increased concentrations of collagen, the neonatal myocardium is less compliant, with shorter filling times and an increased dependency of diastolic function on atrial contraction (23).

Diastole can be divided into two different phases, namely, early and late diastole. The complex distribution and orientation of the different myocardial muscle layers are key anatomic factors that explain not only the highly efficient pumping of blood during systole but also the mechanisms of ventricular filling in early diastole. During IVRT, the clockwise relaxation of the transverse myocardial band and the cessation of contraction of the inner descending fibers, together with the contraction of the outer ascending layer, generate the powerful suction force that is critical for the rapid early filling of the LV after mitral valve opening (24). We found significantly increased IVRT' in both ventricles and the septum, even after correcting for HT-induced bradycardia, that improved progressively during treatment and normalized after rewarming. This finding suggests globally prolonged myocardial relaxation. IVRT' is a good non-invasive index of diastolic function that correlates well with invasive measurements of Tau, the time constant of ventricular relaxation, and its prolongation is associated with diastolic dysfunction (25, 26). Delayed relaxation has been shown to be an important contributor to heart failure with preserved EF (27). This can be explained by the fact that 50–60% of diastolic recoil occurs during IVRT and an intact myocardial untwisting is necessary for an adequate early diastolic function (28, 29). Moreover, 70% of ventricular filling takes place during early diastole. In agreement, prolonged IVRT' in our study was accompanied by a slower early myocardial diastolic velocity (e') in the RV, although not in the septum or the LV and longer RV FT'. TDI e' is a good indicator of ventricular relaxation that correlates well with invasive measurements (30, 31). Altered early filling affecting only the RV is in accordance with the observation of recent studies suggesting a particular vulnerability of the RV to perinatal hypoxia-ischemia (2, 3). A study on normothermic asphyxiated neonates describes biventricular prolongation of IVRT with slower e' only in the RV, but this was not interpreted as a representative of diastolic dysfunction (11).

Rapid early ventricular filling is followed by a transient decrease in flow (diastasis) after which atrial contraction collaborates to a further increase in ventricular filling during the late phase of diastole. Traditionally, relaxation parameters are used to characterize early diastole, whereas compliance and filling pressure parameters are used to characterize late diastole (8). Slow relaxation can limit filling and increase end-diastolic pressures when relaxation remains incomplete at the end of diastole (27). However, although early diastole was affected in our babies, late diastolic myocardial velocities seemed to be preserved during TH, except for a decreased septal a' in T2 that normalized after rewarming. Similarly, no differences were found for E'/A' ratio between babies with HIE and controls. In addition, ventricular compliance, as assessed with the E/e' ratio, remained unchanged, although this should be interpreted with caution. E/e' ratio correlates well with pulmonary capillary wedge pressure and provides a good estimation of ventricular filling pressure independent of relaxation or LV systolic function, and it has been evaluated in neonates in different contexts (3, 6, 9, 16, 30, 32–34). However, E/e' may be influenced by left-to-right shunts through the patent ductus arteriosus (PDA) or foramen ovale (FO), as probably happened in our study, so its role in neonates awaits further clarification (7). The influence of TH may explain the discrepancy between our findings regarding early and late diastole. As recently shown in experimental studies in adult pigs, time for complete ventricular relaxation (3.5 × Tau) may be within diastolic duration during TH despite the presence of diastolic dysfunction due to decreased spontaneous HRs (7, 27, 35, 36). This could explain why delayed relaxation and slowed early filling did not translate into increased filling pressures in our patients. However, in those animal models, if HR is increased with atrial pacing, the duration of diastole is shortened compromising filling (7). This situation may render the myocardium more vulnerable to moderate increases in HR as it happens with the use of inotropic drugs (up to 85.7% in our series). Interestingly, those experimental data as well as clinical studies in adults demonstrate that TH *per se* can induce diastolic dysfunction (37, 38). However, our results are consistent with those of previous studies in asphyxiated neonates not treated with TH, so hypoxia-ischemia seems to play a key role in the development of this condition in these babies (11, 20).

Prolongation of isovolumic relaxation can also be the result of alternative mechanisms, such as altered twist/untwist motion or intraventricular dyssynchrony, both of which impact early diastolic suction (39). In this regard, we found a prolongation of IVCT' during TH, even after correcting for HR, that normalized after rewarming. These results are in agreement with previous studies in non-cooled babies with HIE (11). IVCT' correlates with peak +dP/dt and is prolonged in systolic dysfunction (25). This fact may exacerbate diastolic dysfunction as impaired ventricular twisting may delay untwisting, reduce suction, and impair early diastolic filling (24). Moreover, coordinate systolic contraction itself is a major determinant of early diastolic ventricular filling, so dyssynchrony may have a negative impact on overall ventricular performance leading to prolonged contraction, as well as delayed and prolonged relaxation (40). t-IVT and MPI are sensitive indexes of ventricular dyssynchrony (40), and both parameters were globally prolonged in our study. Some studies have suggested that the increased post-ejection isovolumic period observed in patients with pulmonary hypertension can be explained by prolonged RV contraction beyond pulmonary valve closure due to increased afterload, and not by delayed isovolumic relaxation, and consequently, it does not reflect diastolic dysfunction in this context (41). This mechanism could partly explain the prolongation of IVRT' in our study, considering that PVR was significantly increased in the HIE + TH group (refer to Supplemental Material). However, the slower RV e' in our patients supports the presence of diastolic dysfunction. Besides, LV free wall IVRT' was also prolonged in our series.

Our study has some limitations. First, most patients were receiving dobutamine during the echocardiographic assessments and this could have influenced our results. In this regard, some studies have reported an improvement in diastolic function with this treatment (42). Thus, the frequent use of dobutamine in our series could have modified diastolic function and does not allow us to differentiate whether the progressive improvement observed in our patients was due to the use of inotropes or to spontaneous recovery. However, part of this improvement could also be attributed to the increased use of iNO in T2, as this drug could have improved myocardial function by reducing RV afterload. Dobutamine may also be responsible for the relatively high HR of some of our patients during TH. Furthermore, we could not discriminate whether the observed effects on diastolic function are the result of HIE, TH, or a combination of both factors. Finally, the retrospective design and the small number of patients evaluated may limit the extrapolation of our findings.

In conclusion, our study demonstrates the presence of diastolic dysfunction in neonatal HIE during TH characterized by globally prolonged IVRT' and decreased early diastolic RV myocardial velocity, regardless of HR, which normalized after rewarming, reflecting a prolonged and slowed myocardial relaxation. We also found data, suggesting an altered myocardial twist/untwist motion and ventricular dyssynchrony. Diastolic impairment may not be clinically evident in the setting of hypothermia-induced bradycardia, so echocardiographic assessment of myocardial function during TH should include the evaluation of diastolic function with appropriate techniques, such as TDI. Further studies are needed to investigate the impact of diastolic dysfunction on HIE as well as its potential therapeutic implications.

## Data Availability Statement

The raw data supporting the conclusions of this article will be made available by the authors, without undue reservation.

## Ethics Statement

The studies involving human participants were reviewed and approved by Ethics Committee in Clinical Research, Hospital Clinico San Carlos, Madrid, Spain. Written informed consent to participate in this study was provided by the participants' legal guardian/next of kin.

## Author Contributions

LA and MJR: study design. MJR, AC, and LA: acquisition of data. JM-O and IS: data analysis. MJR, JM-O, IS, and LA: interpretation of data. MJR, LA, and JM-O: manuscript drafting. All authors contributed to the article and approved the submitted version.

## Conflict of Interest

The authors declare that the research was conducted in the absence of any commercial or financial relationships that could be construed as a potential conflict of interest.

## Publisher's Note

All claims expressed in this article are solely those of the authors and do not necessarily represent those of their affiliated organizations, or those of the publisher, the editors and the reviewers. Any product that may be evaluated in this article, or claim that may be made by its manufacturer, is not guaranteed or endorsed by the publisher.
