# Supplementary material for: Diastolic Dysfunction in Neonates With Hypoxic-Ischemic Encephalopathy During Therapeutic Hypothermia: A Tissue Doppler Study
Source: Front Pediatr. 2022 May 25;10:880786. doi: 10.3389/fped.2022.880786 (PMC9174686; doi:10.3389/fped.2022.880786)
Supplement: Supplementary file 1 [file Data_Sheet_1.docx]

Supplementary Material

Conventional echocardiography

Methods:

Fractional shortening (FS) and ejection fraction (EF) were calculated with M-mode from the parasternal long axis view. Tricuspid annular planar systolic excursion (TAPSE) was measured with M-mode from a four-chamber apical view. Early (E) and late (A) tricuspid and mitral valve inflow velocities and their ratio (E/A) were measured with pulsed-wave Doppler from a 4-chamber apical view. Superior vena cava flow (SVCF) was calculated using the velocity time integral (VTI) of the SVCF pulsed Doppler recording obtained from a subcostal view and the diameter (D) of the vessel from a high parasternal long axis view [(SVCF=VTI x π x D2/4 x HR)/weight] (1). Aortic valve D was measured from a parasternal long axis view and aortic flow VTI was obtained with pulsed Doppler from an apical four-chamber view to calculate left ventricular stroke volume [SVLV=(VTI x π x D2/4) /weight] and left ventricular output [LVO=(VTI x π x D2/4 x HR)/weight]. Similarly, pulmonary valve D and VTI were measured from a parasternal long axis view to calculate right ventricular stroke volume [SVRV=(VTI x π x D2/4) /weight] and right ventricular output (RVO= VTI x π x D2/4 x HR)/weight). Pulmonary vascular resistance (PVR) index was calculated using pulsed Doppler images of the right ventricular outflow tract with the formula PVR index = RVET/TPV, where TPV is pulmonary artery time-to-peak velocity.

Results:

SVCF, RVO and SV_RV were significantly reduced at first assessment in the HIE+TH group compared to controls and improved significantly in subsequent evaluations (Table 6). However, SV_RV was still significantly lower at T3 compared to the control group. No differences were found for LVO and SVLV between groups and timepoints, except for an increase in SVLV at T2 in HIE+TH compared to controls. EF and FS were lower at first assessment in HIE+TH babies and, although both improved at T2, they decreased significantly after rewarming compared to controls. TAPSE was also significantly lower in HIE+TH group at T1 as compared to controls but differences disappeared in the following evaluations. MPIRV remained increased at all timepoints in HIE+TH babies compared to controls despite a significant improvement at T2 and T3 compared to baseline values. Similarly, MPILV was significantly increased at T1 and T2 in asphyxiated babies compared to controls but remained unchanged during therapeutic hypothermia and rewarming. Finally, RVET/PAAT was significantly increased compared to controls at all timepoints.

References

1. Kluckow M, Evans N. Superior vena cava flow in newborn infants: a novel marker of systemic blood flow. Arch Dis Child Fetal Neonatal Ed 2000;82:182-7.

Table 6. Conventional echocardiographic measurements.

|  | (Controls T1) | (Controls T2) | (Controls T3) | (HIE+TH T1) | (HIE+TH T2) | (HIE+TH T3) |
| --- | --- | --- | --- | --- | --- | --- |
| **HR** | 129 (124.5-133.5) | 136 (128-146) | 133 (123-137) | 101 (92-124)* | 119.5 (106.8-128.8)* | 137 (128.5-149.5)# |
| **SVCF** | 111.19 (86.93-199.24) | 140.22 (102.51-162.58) | 134.89 (118.45-167.32) | 53.17 (35.58-135.11)* | 108.33 (78.9-133.72)# | 105.13 (78.43-136.02)# |
| **RVO** | 279.94 (256.42-336.39) | 295.78 (261.11-340.9) | 330.87 (284.73-360.07) | 153.52 (115.38-236.74)* | 285.08 (224.13-319.17)# | 268.66 (220.69-355.67)# |
| **SV_RV_** | 2.2 (1.96-2.74) | 2.26 (1.92-2.54) | 2.37 (2.02-2.84) | 1.61 (1.3-1.91)* | 2.22 (1.93-2.66)# | 1.88 (1.63-2.59)*# |
| **LVO** | 171.59 (151.8-209.82) | 170.29 (136.26-213.92) | 182.75 (152.02-226.9) | 143.92 (89.72-245.91) | 191.9 (154.41-252.04) | 182.88 (138.92-256.38) |
| **SV_LV_** | 1.37 (1.25-1.47) | 1.41 (1.16-1.48) | 1.43 (1.23-1.56) | 1.3 (0.81-2.23) | 1.67 (1.3-2.1)* | 1.39 (1.01-1.87) |
| **EF** | 69.8 (67.1-78.1) | 69.9 (67.3-72.5) | 71.3 (68.9-74.1) | 62.6 (52.7-70.1)* | 70.2 (65.2-75.9)# | 60.9 (59.00-72.75)* |
| **FS** | 36.7 (35.1-43.6) | 36.8 (34.8-38.8) | 38.2 (36.1-40.00) | 31.3 (25.00-36.7)* | 37.5 (33.9-42.3)# | 30.4 (28.8-38.7)* |
| **TAPSE** | 0.95 (0.78-1.08) | 0.95 (0.81-1.02) | 0.89 (0.84-1.00) | 0.7 (0.46-0.88)* | 0.83 (0.77-0.92) | 0.88 (0.78-0.96) |
| **MPI_RV_** | 0.19 (0.12-0.27) | 0.15 (0.09-0.26) | 0.1 (0.07-0.12) | 0.43 (0.34-0.52)* | 0.36 (0.28-0.4)*# | 0.24 (0.19-0.41)*# |
| **MPI_LV_** | 0.21 (0.18-0.28) | 0.24 (0.19-0.26) | 0.24 (0.18-0.29) | 0.38 (0.3-0.5)* | 0.32 (0.25-0.4)* | 0.3 (0.25-0.34) |
| **RVET/PAAT** | 2.97 (2.69-3.27) | 2.56 (2.19-2.67) | 2.5 (2.15-2.8) | 4.17 (3.29-5.33)* | 4.02 (3.37-5.06)* | 3.45 (2.75-3.63)* |

Data are expressed as mean (95% CI) or absolute number (%), as appropriate. HIE: hypoxic-ischemic encephalopathy; T1, T2, and T3: timepoints 1, 2 and 3 respectively; HR: heart rate; SVCF, superior vena cava flow; RVO, right ventricular output; SV_RV_, stroke volume of the right ventricle; LVO, left ventricular output; SV_LV_, stroke volume of the left ventricle; EF, ejection fraction; FS, fractional shortening; TAPSE, tricuspid annular plane systolic excursion; MPI_RV_, myocardial performance index of the right ventricle; MPI_LV_, myocardial performance index of the left ventricle; RVET/PAAT: right ventricular ejection time/pulmonary artery acceleration time.

* p<0.05 vs. Control (same time point)

# p<0.05 vs. timepoint 1 (within each group)
